# Perceptions and Attitudes Regarding Postpartum Depression on Reddit: Inductive Content Analysis

**DOI:** 10.2196/105765

**Published:** 2026-10-13

**Authors:** Ananta Srivastava, Farah Mawlawi, Anita Nelson

**Affiliations:** 1 Western University of Health Sciences, 309 E. Second Street, Pomona, CA, 91766, United States

**Keywords:** postpartum depression, PPD, Reddit, social media, inductive content analysis, treatment recommendations, treatment barriers, peripartum

## Abstract

**Background:**

Over the past decade, postpartum depression (PPD) diagnoses have significantly risen across all ethnic and racial groups. However, PPD remains underdiagnosed due in part to stigmatization and misunderstanding by the public. This can result in a reluctance among women to openly discuss their symptoms with clinicians. However, social media offers supportive communities and unique platforms for individuals to openly share the experiences and opinions that they may be less likely to disclose in person. Despite the availability of new Food and Drug Administration (FDA)–approved medical therapies for PPD over the past 7 years, research investigating comments about perceptions of recommendations for and barriers to treatment has been sparse.

**Objective:**

The objective of this study was to evaluate the themes that emerge from comments publicly posted on Reddit discussions regarding PPD to assess perceptions of treatment recommendations and potential treatment barriers.

**Methods:**

A qualitative study was conducted of comments made on Reddit responding to eligible posts published in English between August 2019 and August 2024. These posts were retrieved using the following individual keyword searches: “Zulresso,” “Zurzuvae,” “brexanolone,” “zuranolone,” “PPD,” and “postpartum depression.” Eligible posts had to meet the threshold of 5 or more votes and 5 or more comments. All comments that replied to each selected post were retrieved through the keyword search and Reddit’s default “Relevance” display setting. Within our 2 overarching categories of interest (treatment recommendations and potential barriers to treatment), comments were selected as our main dataset and assigned to a single theme that best matched their primary focus using inductive content analysis.

**Results:**

Among the eligible 93 identified posts, a total of 3482 comments were evaluated for study inclusion. Percentages represent proportions of coded comments. Of those, 1348 comments were selected for content analysis based on relevance to the 2 topic categories of interest; 866 comments discussed treatment recommendations and 482 comments discussed perceived potential treatment barriers. In the category of treatment recommendations, the following themes were identified: support of medication use (450/866, 52%), support for counseling (148/866, 17.1%), recommendation for lifestyle changes (145/866, 16.7%), and support for combination of therapies (123/866, 14.2%). Comments on barriers yielded 4 additional themes: perceived overdiagnosis of PPD and medicalization of situational postpartum distress (187/482, 38.8%), lack of social support and understanding within personal networks (154/482, 32%), insufficient institutional support (97/482, 20.1%), and inadequate health care provider support (44/482, 9.1%).

**Conclusions:**

Our analysis of the Reddit comments revealed that there were significant perceived unmet needs regarding recognition and treatment of PPD, including the optimization of individualized treatment plans, greater public awareness about the condition, and enhanced support from both social and health care networks.

## Introduction

Perinatal depression, which includes postpartum depression (PPD), is a major depressive disorder characterized by persistent feelings of sadness, anxiety, and loss of interest in activities that start during pregnancy or within 4 weeks of childbirth [1]. It represents a generalization of the earlier concept of PPD that focused on the symptoms presenting after delivery and highlighted the fact that both pregnancy and delivery can precipitate them. The American College of Obstetricians and Gynecologists (ACOG) clinical practice guidelines mention that 1 in 7 women report symptoms severe enough to support the diagnosis of perinatal depression, with onset occurring during pregnancy in 33% and postpartum in 40% [2]. More specifically, according to the US Centers for Disease Control and Prevention, the prevalence of self-reported PPD symptoms is 13.2% nationally, ranging from 9.7% to 23.5% depending on the state [3]. Another study reported that PPD rates rose from 9.4% in 2010 to 19% in 2021; the largest increases were observed among Asian and Pacific Islander participants (280% increase) and non-Hispanic Black participants (140% increase) [4]. While rising PPD rates may indicate improved screening and diagnosis, especially in marginalized groups, these statistics only represent diagnosed cases. It is estimated that up to 50% of mothers with PPD go underdiagnosed by their health care providers [4].

Early diagnosis is critical as approximately one-quarter of women with perinatal depression continue to experience symptoms up to 3 years after delivery [5]. If untreated, PPD can have significant adverse maternal and infant outcomes. Maternal consequences include impaired mother-infant bonding, reduced initiation and duration of breastfeeding, poor relationships, decreased adherence to medical care, and increased maternal morbidity [6]. In severe cases, untreated PPD is associated with an elevated risk of maternal suicide and, rarely, infanticide [6]. For the offspring, exposure to maternal PPD is linked to poorer social-emotional functioning, delays in developmental milestones, and impairments in adaptive behavior [7]. There is evidence suggesting that these adverse effects are not limited to infancy but may persist into later childhood and adolescence, contributing to long-term neurodevelopmental and behavioral difficulties [4].

Managing this condition, including counseling patients and their families about treatment recommendations, falls within the scope of practice for obstetrician-gynecologists and other perinatal health care providers [8]. Current first-line treatments for PPD include psychotherapy, pharmacotherapy, or a combination of both [8]. Psychotherapy is generally the first-line therapy for mild to moderate cases, but accessibility and patient preference vary widely. The ACOG recommends selective serotonin reuptake inhibitors as the first-line pharmacotherapy for perinatal depression but notes that serotonin-norepinephrine reuptake inhibitors are also reasonable alternatives [8]. For previously untreated patients, escitalopram or sertraline are generally recommended [8]. For moderate to severe depression with onset in the third trimester or within 4 weeks post partum, the ACOG originally recommended the neurosteroid brexanolone, which was the first Food and Drug Administration (FDA)–approved medication specifically for PPD [8,9]. As brexanolone was commercially discontinued in 2024, zuranolone is indicated for treatment in these situations [10].

Despite the availability of effective treatments and recent treatment advancements, many women continue to face barriers to diagnosis and care. Previous researchers have identified barriers to treatment engagement including lack of time, transportation, childcare, and financial constraints [1]. Stigmatization surrounding PPD is another limiting factor for patients seeking care or accepting treatment. Having this condition can be seen as a personal deficit or failure as a mother and promotes fear of abandonment and lack of support upon diagnosis [5]. Consequently, this has also contributed to women being reluctant to openly discuss their symptoms [11,12]. In addition, health care system shortcomings have been found to restrict women’s ability to access care [13].

As a result of these barriers, many women seek support outside traditional health care settings. Social media has emerged as a valuable research platform for understanding the lived experiences of those with health conditions that are frequently stigmatized or underdiscussed in clinical settings [14,15]. Perhaps because of the anonymity of those platforms, women with PPD may be more willing to disclose their emotional distress, treatment hesitancy, and perceived barriers to care, which might otherwise remain unreported [16]. Hence, women with PPD have increasingly turned to online platforms because of their potential to offer supportive communities and validation from peers with shared experiences [17].

Although social media has been used to study PPD, only a few studies have explored women’s attitudes toward treatment and barriers to care for PPD within these new online environments. Prior studies have used online surveys of women with PPD and their partners to help identify risk factors and markers for the condition [18-21]. Other studies have explored social media sites as a source of patient information [22]. Additional studies from China have explored social support and nonadaptive communication styles used by family members associating with women who have PPD [23,24].

This study leveraged social media to address a different aspect of PPD from what has been previously explored. We studied the themes expressed in spontaneous, user-generated comments about PPD treatments and potential barriers responding to eligible posts on the topic.

## Methods

### Study Design and Population

This study followed a qualitative, retrospective design using an inductive approach to analyze public discussions in English about treatments for and barriers to PPD treatment on Reddit posts published from August 2019 to August 2024. The search was conducted on August 21, 2024, using a newly created Reddit account shared by all reviewers for the sole purpose of this study. Posts were identified through a search using the following keywords: “Zulresso,” “Zurzuvae,” “brexanolone,” “zuranolone,” “PPD,” and “postpartum depression.” The keywords were entered into the search bar as individual search queries without the use of Boolean operators. The search was conducted across Reddit generally rather than within specific subreddits. Posts identified through the search originated from various subreddits, including r/Postpartum_Depression, r/BabyBumps, r/NewParents, r/beyondthebump, and r/parenting.

### Inclusion and Exclusion Criteria

Data collection was initiated by inputting keywords into Reddit’s search feature, which offers multiple options for organizing how posts are displayed, including “Relevance,” “Hot,” “Top,” “New,” or “Comment count.” Of these built-in platform options, the “Relevance” display setting was used to populate the posts. Posts retrieved through this search were included if they met 2 criteria: at least 5 votes and at least 5 comments. No secondary or post hoc filters were applied at the post level, ensuring that every comment from an eligible post was considered.

All available comments and nested replies associated with eligible posts were evaluated for inclusion. Comments were included if they were considered evaluable content that was relevant to either treatment recommendations or perceived barriers to PPD treatment. Comments that were irrelevant to these categories, comments only including brief words of support without content, and automated or bot-generated comments were excluded. Deleted, removed, and verbatim duplicate comments from the same source were also not included. Supportive comments containing substantive discussion of treatment recommendations or perceived barriers were retained. As all eligible comments meeting the previously mentioned criteria were included regardless of whether multiple comments were from the same user, thematic saturation was not used. These comments served as the primary dataset for inductive content analysis.

### Data Extraction

The comments that made it through the inclusion and exclusion criteria filters were manually extracted by 2 reviewers and pasted from each post into a Google spreadsheet. No API or automated data retrieval programs were used. Data extraction occurred within approximately 1 to 2 months following the August 21, 2024, search. The sheet listed the following metrics of interest: comment, Reddit user or commenter, number of upvotes, and designated theme.

### Qualitative Analysis

All included comments were analyzed using inductive content analysis. The analysis was conducted by 2 medical student reviewers. Neither reviewer had formal training in qualitative research methods, but they followed up with their principal investigator as needed. Rather than applying predetermined categories, codes, and categories were developed inductively from the data.

To establish a baseline understanding and consistency between the 2 researchers, they coded a preliminary subset of approximately 20 comments together. Once a mutual agreement on coding methodology was achieved from the initial sample, both researchers then independently conducted an inductive content analysis of all remaining comments. The coding framework was iteratively refined throughout the analysis as new concepts emerged. Approximately every 50 comments, the reviewers revisited the existing categories and discussed whether they adequately represented the data or required modification.

Each comment was assigned to a single theme that matched its primary focus to ensure that comments were counted only once, and thematic frequencies reflected mutually exclusive categories. If a comment contained content relevant to more than one theme, it was assigned to the theme that was most emphasized. Themes were grouped into 2 major categories of interest: those that referred to treatment recommendations and those that referred to barriers that the commenters perceived as impeding access to care.

At the conclusion of the coding process, the comments were collectively reviewed by both researchers, and any inconsistencies, including uncertainty regarding the primary theme of multi-topic comments, were discussed until an agreement was reached. Formal intercoder agreement statistics were not calculated because the analysis used an iterative consensus-based approach. Independent coding and consensus review were used to support the credibility and dependability of the coding process. The researchers recognize that qualitative interpretation may be influenced by researcher perspectives, but this process was used to reduce the influence of any single researcher’s interpretation. Themes were inductively grounded in comment data and supported by illustrative quotes to enhance confirmability. The comment was the unit of analysis, and all reported frequencies and percentages represent the proportion of coded comments rather than unique users or individuals.

### Ethical Considerations

This study relied exclusively on publicly available online content and did not involve any interaction with Reddit users. Selected quotes were left incomplete to limit searchability and in an effort to preserve the anonymity of commenters. A random selection of quotations was searched online to assess whether the text could be traced to the original Reddit posts, and quotations were shortened or paraphrased when appropriate to reduce reidentification risk. The Western University of Health Sciences Institutional Review Board reviewed the project (reference 24RFD039) and deemed that this study did not constitute human subject research.

## Results

### Data Analysis

Initially, keywords identified 386 Reddit posts, of which 93 met the inclusion criteria. These 93 posts had 3482 comments, from which 2134 were excluded because they only offered brief supportive words without content or irrelevant content. From the remaining 1348 comments included in the inductive content analysis, 64.2% (n=866) discussed treatment recommendations, whereas the remaining 35.8% (n=482) focused on perceived barriers to treatment. Figure 1 illustrates a study flow diagram of this progression. Table 1 reveals the 4 major themes from the comments that discussed treatments. Medication use was the most frequently coded treatment recommendation, accounting for 52% (450/866) of treatment-related comments. Table 2 summarizes the major themes that emerged about potential treatment barriers. The most common theme in this category was perceived overdiagnosis of PPD and medicalization of situational postpartum distress. Finally, the following paragraphs delve into the detailed findings of the content analysis, with Tables 3 and 4 providing more complete examples of the comments that were classified in each theme.

**Figure 1.**
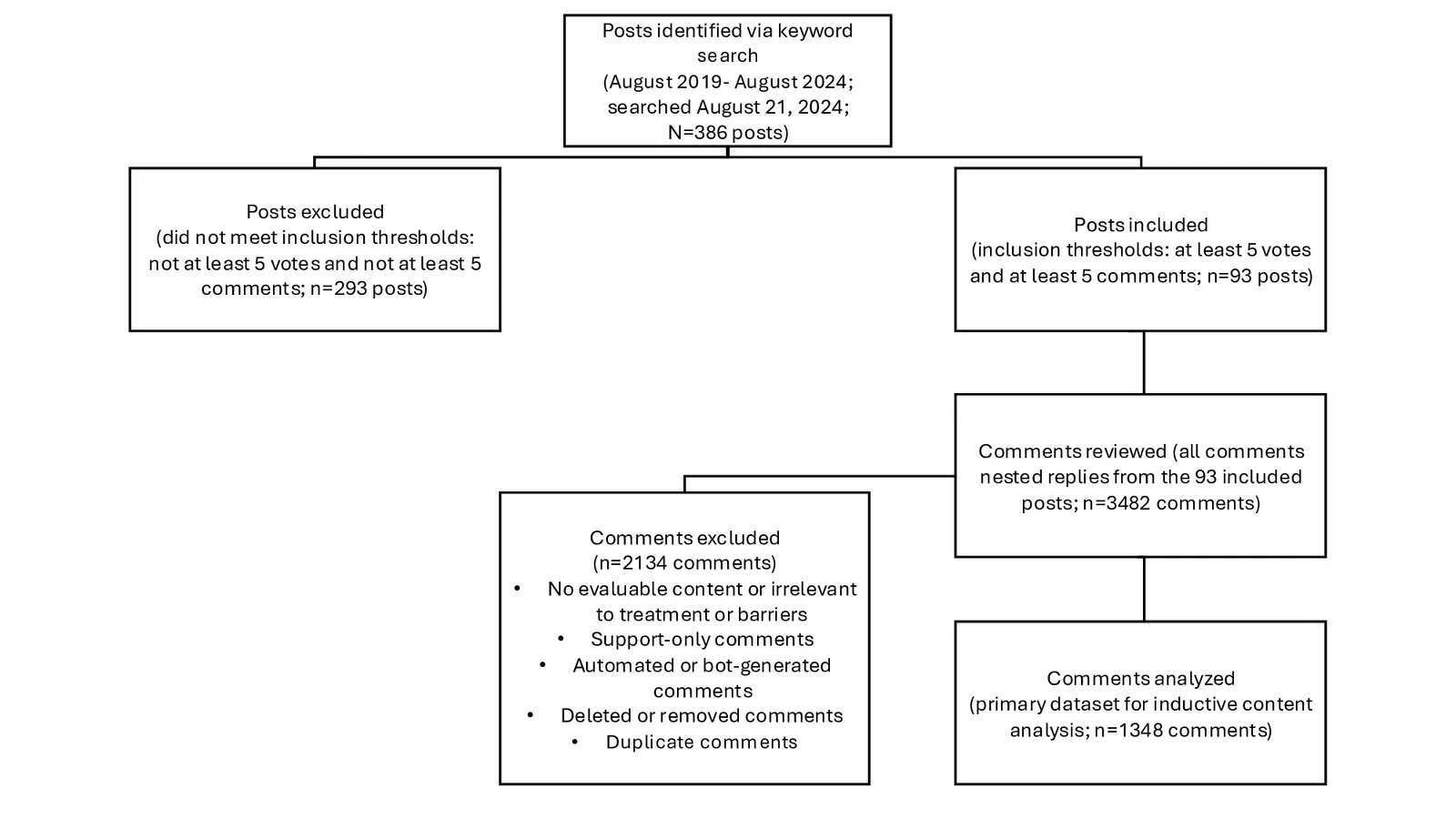
Study flow diagram showing the progression of Reddit posts and their associated comments through the screening and inclusion process.

**Table 1.** Frequency of comments for key themes regarding treatment recommendations based on personal experiences (n=866 comments).

Theme	Comments, n (%)
Medication use	450 (52)
Counseling	148 (17.1)
Lifestyle changes	145 (16.7)
Combination of therapies	123 (14.2)

**Table 2.** Frequency of comments for key themes regarding treatment barriers (n=482 comments).

Theme	Comments, n (%)
Patient perception of overdiagnosis of PPD^a^ and medicalization of situational postpartum distress	187 (38.8)
Lack of social support or understanding in personal networks	154 (32)
Lack of institutional support	97 (20.1)
Lack of adequate health care provider support	44 (9.1)

a PPD: postpartum depression.

**Table 3.** Selected Reddit comments from each of the identified subthemes under treatment recommendations for postpartum depression.

Themes	Illustrative comments
Support for medication use	“...when I was deeply struggling...I would be reading books to my baby and sobbing etc ...I felt like that antidepressants really did make me feel better. It’s not always getting to the bottom of the why, but just trying to improve the way you feel now.” “I was prescribed Zoloft about 2 or 3 months PP [postpartum]. My only regret is not starting it sooner!” “I had severe PPD with both pregnancies.... My OB referred me for Zulresso infusion ...It helped me immensely! I went from suicidal to almost back to normal.”
Support for counseling	“...Talk to someone about it!.... Acknowledging the issue is a big step. You can and will heal, it’s an absolute myth that you can’t. You’ll look back...and feel blessed to have gone through this journey.” “Your healthcare provider is there to help you. You...can talk about feeling low and/or anxious and ask for help.... Getting help is the best thing you can do...”
Support for lifestyle changes	“...it can help to change up something major. Workplace, new friends, hobbies, states, whatever.” “...Making a significant lifestyle change could...address multiple triggers at once.”
Support for combination of therapies	“If u find yourself drowning ask for help! Friends, family and tell them what u need. I know it’s hard.... Therapist and meds helped me with my PPA/PPD. Then marriage therapy! It’s been a rollercoaster for us!” “I felt that way during my post partum depression... With counseling and a low dose antidepressant for sleep. And time. Also eating and drinking and meeting my needs more and realizing I shouldn’t feel guilty about...showering and taking breaks.”

**Table 4.** Selected Reddit comments from each of the identified subthemes under barriers to treatment for postpartum depression (PPD).

Themes	Illustrative comments
Perception of PPD overdiagnosis and medicalization of situational postpartum distress	“And there will STILL people who will be like ‘Maybe it’s PPD! You should go on meds and see a therapist!’.... I bet if you took any person (man or woman) and put them in some of these situations, they would have a mental health break as well.” “100% agree. I do not doubt that ppd/ppa exist but the way people casually slap these diagnoses around is reminiscent of the old diagnoses of ‘hysteria’ etc. It’s those womanly chemicals that are oUt oF cOnTrOl...not the assholes in your life and the physical pain and the sleep deprivation and etc.”
Lack of social support and understanding within personal networks	“Families and communities failing to show up as a village.... There’s no acknowledgement that sometimes, that village disappears, doesn’t want to help, or simply never existed. [There needs to be more] listening, troubleshooting, and helping parents adjust in a way that maintains their identity outside of parenthood.... I see women gaslit and even gaslighting themselves constantly [into thinking they] need to be medicated...but NOT saying that maybe their husbands need to step up because mom having 0 minutes of personal time...isn’t okay or sustainable” “I am fully convinced that half the cases of PPD [involve] lack of support for the mother and the overwhelming stress and exhaustion. I don’t have ‘a village.’ There’s no one I can ask for help”
Inadequate health care provider support	“...my concerns were brushed off even by medical professionals. I finally got the courage to admit that I wasn’t OK”
Insufficient institutional support	“Post partum care in the US is so flippant about woman’s mental (and even physical) health. Once the baby (or babies) are out it’s just bye bye you got this!” “We get ONE PP appointment. That’s not sufficient.”

### Category 1: Themes Related to Treatment Recommendations

Category 1 consisted of 64.2% (866/1348) of total comments.

#### Support for Medication Use

In response to posts concerning PPD, 52% (450/866) of the comments that discussed treatment recommendations encouraged medication use for management. Specifically, those that described personal experiences using medication emphasized that, in many cases, medication may be necessary to support mental health and framed it as a valid and effective approach to recovery from PPD. For example, one individual shared the following:

Getting medicine isn’t cheating.... Your body is forced to cope with something that tough.... With hormones going nuts, sometimes it screws with your baseline emotional/hormonal state. Meds...can help get to the right chemical state again.

Another comment described that medication improved their day-to-day functioning, sharing that “just being fine isn’t the way to live.”

#### Support for Counseling

Another 17.1% (148/866) of the comments described professional counseling to help manage PPD symptoms and encouraged further clinical evaluation,if needed. Some comments said that if a mother felt more overwhelmed than usual, there was no harm in seeking out counseling, with one stating that “what do you have to lose (for being) checked out by a professional?” Another encouraged seeking out professional help without shame “because it is not how you’re supposed to feel overall all the time.”

#### Support for Lifestyle Changes

Lifestyle adjustments were described in 16.7% (145/866) of the comments as another strategy for managing PPD symptoms. Many attributed worsening of their mood to inadequate rest and difficulty with completing everyday tasks, with noted improvement when they were able to reframe their daily routines to support their own needs. Some were able to accomplish it with the mindset of “my kid fits into my lifestyle, not the other way around.” Others emphasized the role of basic self-care in reducing symptom severity, such as completing small tasks such as putting ice on their face or taking a hot shower. Similarly, when symptoms became overwhelming, some comments suggested environmental changes, such as to “go back home and be with family.”

#### Support for Combination of Therapies

To manage PPD symptoms, 14.2% (123/866) of the comments discussed using a multipronged approach with a combination of medication, therapy, and/or lifestyle changes. Some users wished that they had begun therapy and taking medications earlier to return to their baseline. One described that once she “started the meds and therapy and started to feel better, then the guilt set in that I should have gotten help sooner.” Others emphasized the value of integrating professional help with a balanced routine. One comment illustrated this by noting that “medication and therapy have been helping [as well as] yoga...and [I’m] planning on getting back to the gym.”

### Category 2: Themes Related to Perceived Barriers to Treatment

Category 2 consisted of 35.8% (483/1348) of total comments.

#### Perception of PPD Overdiagnosis and Medicalization of Situational Postpartum Distress

Comments under this theme were characterized by 2 related but distinct concerns: both skepticism about the potential overdiagnosis of PPD and concerns that distress arising from difficult postpartum circumstances may be medicalized without sufficient attention to underlying social, relational, and physical stressors. Over one-third of Reddit comments (187/482, 38.8%) in this category reflected the belief that PPD was diagnosed often too quickly, expressing that there was often an unclear distinction made between normal mood changes postpartum and underlying clinically diagnosed mood disorders. One comment noted that we are “doing ourselves a disservice by overdiagnosis or overtreating these very normal responses to a great change in life.” In a similar manner, another described the perception that those who medically treat PPD were “too quick to diagnose and blame hormones for everything. Normal reactions to stressful situations aren’t pathology.” Furthermore, comments mentioned concerns about the difficulty distinguishing between whether they were truly experiencing PPD or simply the effects of significant situational changes in the postpartum period. One comment questioned the following:

Am I really suffering from PPD or am I just sleep deprived? Can I expect my life to actually improve with treatment, or will that only come with time and (someday) sleep?

#### Lack of Social Support and Understanding Within Personal Networks

Approximately another third (154/482, 32%) of comments discussed how limited emotional validation and limited support from close family and friends impaired their recovery and that those in their social networks often minimized their symptoms and underplayed the severity of their distress. One individual reported that her mother “flat out told me that I had no reason to be depressed.” Additionally, a comment noted that the lack of support could exacerbate vulnerability to PPD as “being unsupported creates the environment that causes PPD/PPA.... We break ourselves trying to do what we are required to do with no help or support.”

#### Insufficient Institutional Support

One in five comments (97/482, 20.1%) reflected insufficient institutional support, describing the perspective that there was a lack of infrastructure to support them and help them recover from PPD. One comment shared that the “systems in place do not set mothers up for health, wellness, and in some cases as mine—basic functioning after having a child.”

#### Inadequate Health Care Provider Support

Approximately 1 in 10 comments (44/482, 9.1%) reflected inadequate health care provider support, reporting that when they were concerned about their symptoms, they had trouble finding professional help. Even when there were clear indicators that help was required, physicians “had no resources given to me despite continually rising scores on my depression surveys at my OB/pediatric appointments.”

## Discussion

### Principal Findings

Given that our search keywords included the use of specific drug names, comments discussing medication were predictably a substantial proportion of our analyzed data. Therefore, the frequency of medication-related comments should not be interpreted as evidence that medication is the preferred treatment among people with PPD. However, it is important to note the remainder that emphasized the role of psychotherapy, counseling, and lifestyle changes. Overall, these comments reflected perceptions of PPD as a multifactorial condition in which pharmacologic and nonpharmacologic strategies are viewed as complementary rather than competing approaches. Among comments that mentioned perceived barriers to care, two related but distinct sentiments emerged. Some expressed skepticism that PPD is overdiagnosed, attributing symptoms to normal postpartum adjustment rather than an underlying mental health condition, whereas others did not dispute the severity of their distress but objected to its framing as a mood pathology, viewing it instead as a medicalization of unaddressed burdens such as inadequate partner involvement, absent support, physical pain, and sleep deprivation. The former may reflect broader skepticism toward psychiatric labeling and has important clinical implications as it can contribute to a delay in or even avoidance of treatment. Additionally, several comments reflected shared experiences with a lack of understanding and support from both social networks and health care providers, underscoring a persistent gap in awareness of and education about PPD. Finally, structural barriers such as limited access to affordable mental health services and inadequate parental leave highlight how systemic constraints intersect with individual beliefs to shape treatment engagement. Taken together, these findings illustrate that online discourse can reflect experiences with PPD but also serves as a space where perceptions of diagnosis and treatment are actively constructed and reinforced.

### Comparison With Prior Work

Zingg et al [25] conducted a multifaceted investigation in which they surveyed care providers and low–socioeconomic status women, as well as conducting a secondary analysis of online posts on PPD from Reddit, Twitter (subsequently rebranded X), and Facebook. They found that women used these social media platforms to share their personal stories about PPD, inquire about the best treatments, vent about family relationships, and provide social support [25]. Our study similarly identified the popularity of those themes within the Reddit comments analyzed but also highlights unmet needs for institutional and professional support. Those themes emerged only from focus groups in the work by Zingg et al [25].

Virani et al [26] followed a study design similar to the one we used by analyzing the comments to YouTube videos about PPD. They found that most conversations focused on the stigma of PPD associated with men and celebrities. They highlighted the perspectives of individuals with PPD and those who stigmatized them, demonstrating how users adapted despite adversity [26]. The findings of our study were consistent with those of Virani et al [26], but our results analyzing the comments from the relevant Reddit posts highlighted additional perceived barriers to care, such as professional indifference, and elucidated how social media can be used as a platform to exchange ideas about effective therapies.

Zhang et al [23] and Zou et al [24] both focused on the specific cultural issues that might be responsible for China’s particularly high rate of PPD. They highlighted the lack of spousal and mother-in-law support, as well as the nonadaptive communication styles used when family talked to those with PPD [23,24]. Our study found that comments involved more widespread criticism of the lack of support and help the users received, but they also emphasized that physicians dismissed their concerns and the health care system itself blocked their access to the health care they thought they needed. Our study also highlights perceptions that PPD may be overdiagnosed.

### Strengths and Limitations

The strengths of this study include the use of Reddit as a valuable platform for mental health research. Compared to formal surveying, Reddit allows for more open, spontaneous discussion about PPD and other stigmatized topics due to its anonymity, discussion format, and availability of topic-specific communities. In particular, the focus on comment-level analysis offers insights into interactive discourse, reflecting how perceptions are shaped within peer communities rather than being only expressed in isolation.

However, the qualitative design of the study and anonymous nature of Reddit, due to which we were unable to verify demographics such as gender and clinical diagnosis of PPD, limited the statistical generalizability to the overall PPD population. Reddit’s user demographics may also differ from those of the broader population experiencing or at risk of PPD, and the analysis was limited to English-language content, which may further limit the generalizability of the findings. Importantly, these findings reflect perceptions expressed in online discussions and cannot establish whether PPD is clinically overdiagnosed. Additionally, comments were nested within posts, and multiple comments may have been posted by the same user. Therefore, comments may not represent independent observations, and reported frequencies should not be interpreted as estimates of the proportion of Reddit users or individuals with a particular view.

There may also have been sampling bias due to the use of Reddit’s default “Relevance” search setting to populate initial posts. As data collection was restricted to this specific parameter without cross-comparing results from alternative platform search options such as “Hot,” “Top,” “New,” or “Comment count,” the exact influence of the platform’s relevance algorithm on the final dataset remains unmeasured. Similarly, our choice of keywords specifically naming certain medications may have overrepresented those treatments and skewed the distribution of comments discussing treatment recommendations toward pharmacotherapy. Additionally, requiring posts to have at least 5 votes and 5 comments may have preferentially selected more popular or highly engaged discussions. These findings may not definitively be reflective of broader or less frequently expressed sentiments regarding preferred treatment modalities.

### Conclusions

Overall, the experiences shared in the comments to posts on Reddit illustrate how this social media platform can provide a space where people can reflect on some of the current constraints of traditional health care. These comments included, but were not limited to, encouraging individualized treatment plans, increasing awareness of PPD, and improving support from social networks and health care systems. Notably, the coexistence of advocacy for multimodal treatment and skepticism toward PPD diagnosis highlights a critical tension that may influence care-seeking behaviors and treatment adherence. The themes identified in these online discussions highlight potential gaps in PPD care and education that warrant further exploration. Furthermore, with the advancement of new medical treatments for PPD, such as neurosteroids, future research should focus on better understanding the experiences with these treatment modalities and broader perspectives shared in social media communities. Ultimately, integrating insights from online communities into clinical practice and public health strategies may offer new opportunities to improve education, communication, and access to PPD care.

## Abbreviations

ACOG: American College of Obstetricians and Gynecologists
FDA: Food and Drug Administration
PPD: postpartum depression

## References

[1] Stewart DE, Vigod S (2016). Postpartum depression. N Engl J Med.

[2] (2023). Screening and diagnosis of mental health conditions during pregnancy and postpartum: ACOG Clinical Practice Guideline No. 4. Obstet Gynecol.

[3] Bauman BL, Ko JY, Cox S (2020). Vital signs: postpartum depressive symptoms and provider discussions about perinatal depression - United States, 2018. MMWR Morb Mortal Wkly Rep.

[4] Khadka N, Fassett MJ, Oyelese Y (2024). Trends in postpartum depression by race, ethnicity, and prepregnancy body mass index. JAMA Netw Open.

[5] Putnick DL, Sundaram R, Bell EM (2020). Trajectories of maternal postpartum depressive symptoms. Pediatrics.

[6] Slomian J, Honvo G, Emonts P, Reginster JY, Bruyère O (2019). Consequences of maternal postpartum depression: a systematic review of maternal and infant outcomes. Womens Health (Lond).

[7] Rogers A, Obst S, Teague SJ (2020). Association between maternal perinatal depression and anxiety and child and adolescent development: a meta-analysis. JAMA Pediatr.

[8] (2023). Treatment and management of mental health conditions during pregnancy and postpartum: ACOG Clinical Practice Guideline No. 5. Obstet Gynecol.

[9] American College of Obstetricians & Gynecologists (2026). Zuranolone and brexanolone for the treatment of postpartum depression. Obstet Gynecol.

[10] (2025). Sage Therapeutics, Inc.; withdrawal of approval of a new drug application for ZULRESSO (brexanolone) solution, 100 milligrams/20 milliliters. Federal Register.

[11] Iturralde E, Hsiao CA, Nkemere L (2021). Engagement in perinatal depression treatment: a qualitative study of barriers across and within racial/ethnic groups. BMC Pregnancy Childbirth.

[12] Carlson K, Mughal S, Azhar Y, Siddiqui W (2025). StatPearls.

[13] Sambrook Smith M, Lawrence V, Sadler E, Easter A (2019). Barriers to accessing mental health services for women with perinatal mental illness: systematic review and meta-synthesis of qualitative studies in the UK. BMJ Open.

[14] Sharma C, Whittle S, Haghighi PD, Burstein F, Sa’adon R, Keen HI (2020). Mining social media data to investigate patient perceptions regarding DMARD pharmacotherapy for rheumatoid arthritis. Ann Rheum Dis.

[15] An MH, Kim MG, Kim J, Chang S, Lee DY, Park RW (2025). Public perception and changing attitudes toward antidepressants over a decade in social media: lessons learned from online discussion using artificial intelligence. PLoS One.

[16] Andalibi N, Ozturk P, Forte A (2017). CSCW ’17: Proceedings of the 2017 ACM Conference on Computer Supported Cooperative Work and Social Computing.

[17] Evans M, Donelle L, Hume-Loveland L (2012). Social support and online postpartum depression discussion groups: a content analysis. Patient Educ Couns.

[18] Andrei AM, Webb R, Enea V (2023). Health anxiety, death anxiety and coronaphobia: predictors of postpartum depression symptomatology during the COVID-19 pandemic. Midwifery.

[19] Trifan A, Semeraro D, Drake J, Bukowski R, Oliveira JL (2020). Social media mining for postpartum depression prediction. Stud Health Technol Inform.

[20] Shatte AB, Hutchinson DM, Fuller-Tyszkiewicz M, Teague SJ (2020). Social media markers to identify fathers at risk of postpartum depression: a machine learning approach. Cyberpsychol Behav Soc Netw.

[21] Gopalakrishnan A, Gururajan R, Venkataraman R (2023). Attribute Selection Hybrid Network model for risk factors analysis of postpartum depression using social media. Brain Inform.

[22] Nguyen QC, Aparicio EM, Jasczynski M (2024). Rosie, a health education question-and-answer chatbot for new mothers: randomized pilot study. JMIR Form Res.

[23] Zhang W, Liu L, Cheng Q, Chen Y, Xu D, Gong W (2020). The relationship between images posted by new mothers on WeChat Moments and postpartum depression: cohort study. J Med Internet Res.

[24] Zou W, Huang L, Yan Y (2024). “It slowly drained the life out of me, leaving me dead inside”: coping and response patterns among Chinese women experiencing postpartum depression. Health Commun.

[25] Zingg A, Singh T, Franklin A (2023). Digital health technologies for peripartum depression management among low-socioeconomic populations: perspectives from patients, providers, and social media channels. BMC Pregnancy Childbirth.

[26] Virani A, Nagra B, O’Mahony J, Bacsu J, Ghatore JK, Panda S (2024). Using YouTube comments data to explore postpartum depression in social media: an infodemiology study. Int J Environ Res Public Health.

